# Supplementary figures and images for: Design of a micro-learning framework and mobile application using design-based research
Source: PeerJ Comput Sci. 2023 Mar 9;9:e1223. doi: 10.7717/peerj-cs.1223 (PMC10280576; doi:10.7717/peerj-cs.1223)

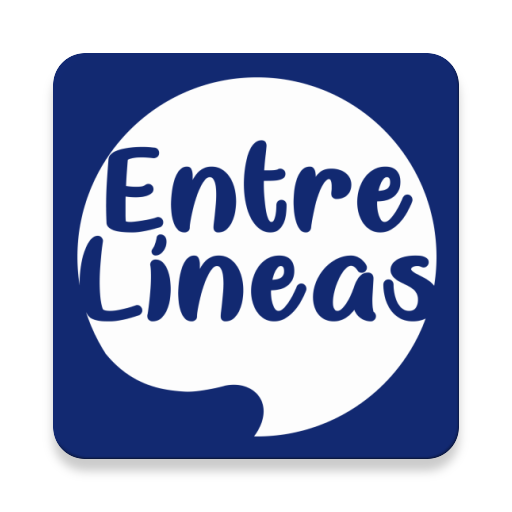

Supplement: Supplemental Information 1 [file peerj-cs-09-1223-s001.zip › BLApp-master/android/app/src/main/ic_launcher-web.png]

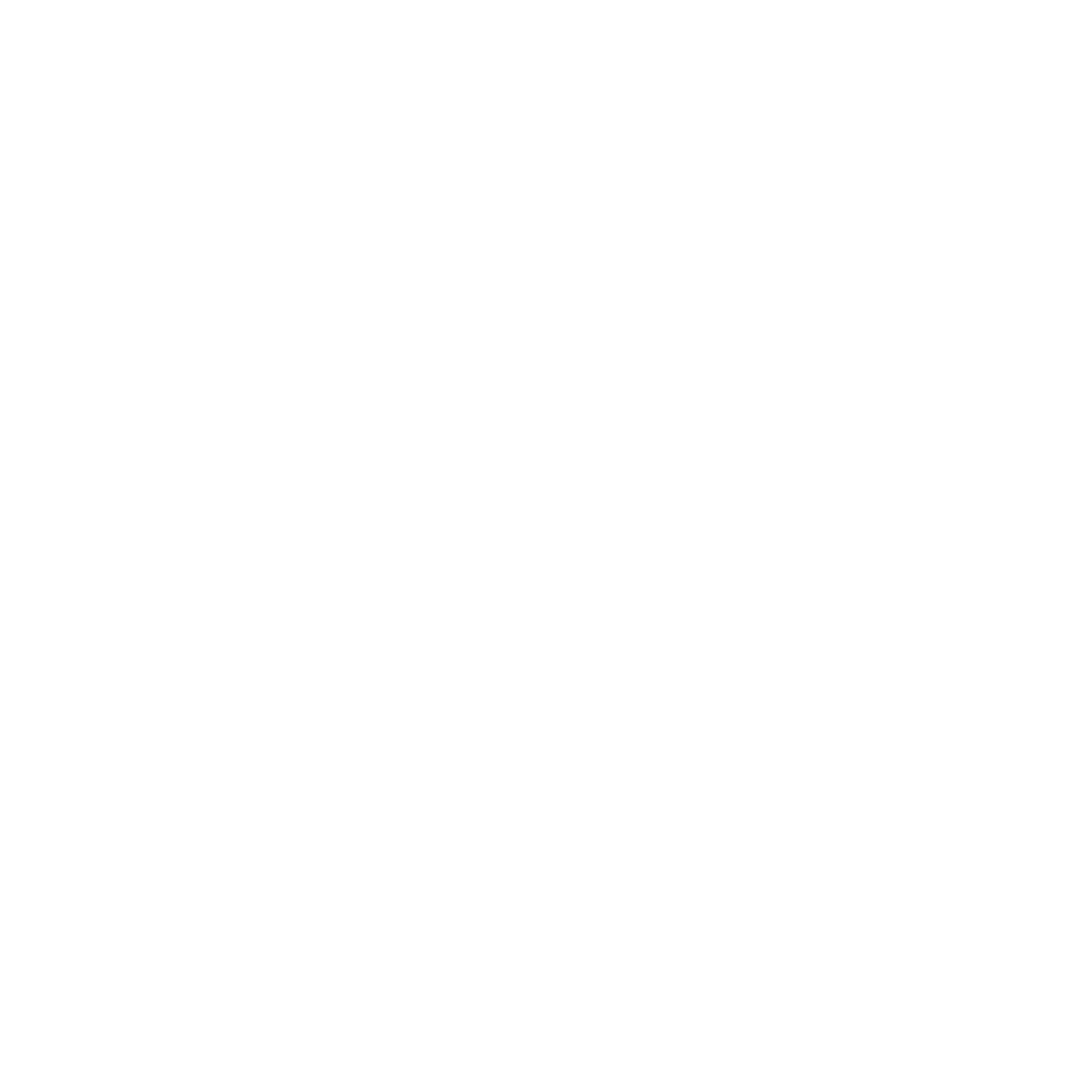

Supplement: Supplemental Information 1 [file peerj-cs-09-1223-s001.zip › BLApp-master/android/app/src/main/res/drawable/logo_app_n.png]

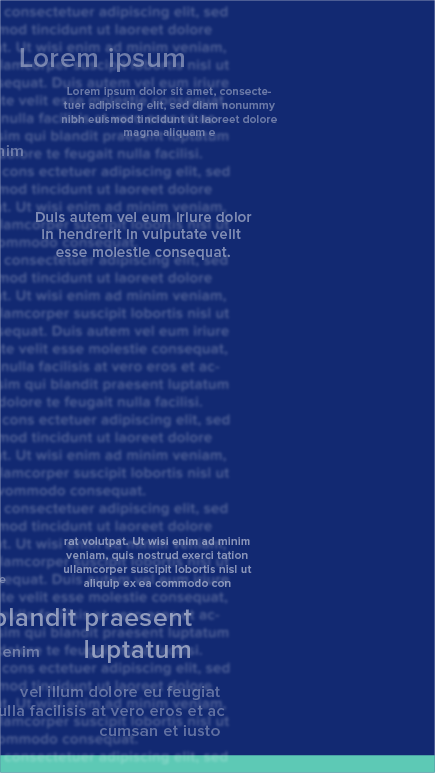

Supplement: Supplemental Information 1 [file peerj-cs-09-1223-s001.zip › BLApp-master/android/app/src/main/res/drawable/splash.png]

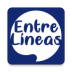

Supplement: Supplemental Information 1 [file peerj-cs-09-1223-s001.zip › BLApp-master/android/app/src/main/res/mipmap-hdpi/ic_launcher.png]

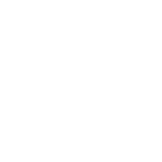

Supplement: Supplemental Information 1 [file peerj-cs-09-1223-s001.zip › BLApp-master/android/app/src/main/res/mipmap-hdpi/ic_launcher_foreground.png]

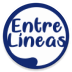

Supplement: Supplemental Information 1 [file peerj-cs-09-1223-s001.zip › BLApp-master/android/app/src/main/res/mipmap-hdpi/ic_launcher_round.png]

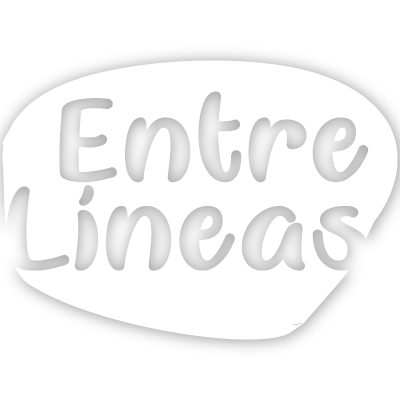

Supplement: Supplemental Information 1 [file peerj-cs-09-1223-s001.zip › BLApp-master/android/app/src/main/res/mipmap-hdpi/icon.png]

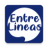

Supplement: Supplemental Information 1 [file peerj-cs-09-1223-s001.zip › BLApp-master/android/app/src/main/res/mipmap-mdpi/ic_launcher.png]

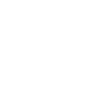

Supplement: Supplemental Information 1 [file peerj-cs-09-1223-s001.zip › BLApp-master/android/app/src/main/res/mipmap-mdpi/ic_launcher_foreground.png]

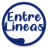

Supplement: Supplemental Information 1 [file peerj-cs-09-1223-s001.zip › BLApp-master/android/app/src/main/res/mipmap-mdpi/ic_launcher_round.png]

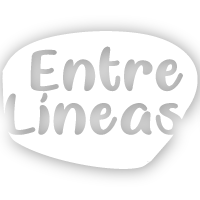

Supplement: Supplemental Information 1 [file peerj-cs-09-1223-s001.zip › BLApp-master/android/app/src/main/res/mipmap-mdpi/icon.png]

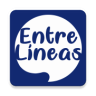

Supplement: Supplemental Information 1 [file peerj-cs-09-1223-s001.zip › BLApp-master/android/app/src/main/res/mipmap-xhdpi/ic_launcher.png]

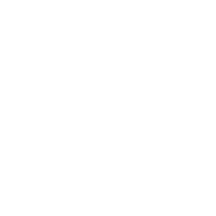

Supplement: Supplemental Information 1 [file peerj-cs-09-1223-s001.zip › BLApp-master/android/app/src/main/res/mipmap-xhdpi/ic_launcher_foreground.png]

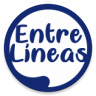

Supplement: Supplemental Information 1 [file peerj-cs-09-1223-s001.zip › BLApp-master/android/app/src/main/res/mipmap-xhdpi/ic_launcher_round.png]

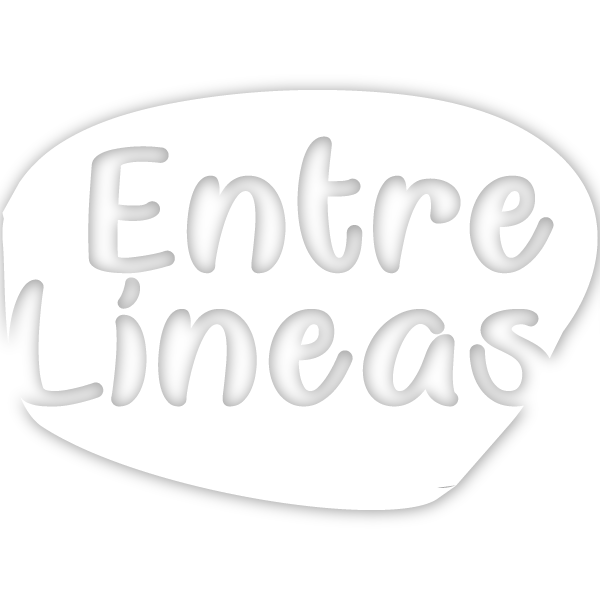

Supplement: Supplemental Information 1 [file peerj-cs-09-1223-s001.zip › BLApp-master/android/app/src/main/res/mipmap-xhdpi/icon.png]

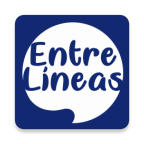

Supplement: Supplemental Information 1 [file peerj-cs-09-1223-s001.zip › BLApp-master/android/app/src/main/res/mipmap-xxhdpi/ic_launcher.png]

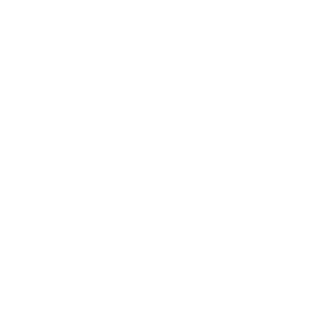

Supplement: Supplemental Information 1 [file peerj-cs-09-1223-s001.zip › BLApp-master/android/app/src/main/res/mipmap-xxhdpi/ic_launcher_foreground.png]

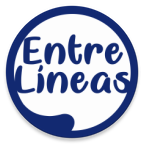

Supplement: Supplemental Information 1 [file peerj-cs-09-1223-s001.zip › BLApp-master/android/app/src/main/res/mipmap-xxhdpi/ic_launcher_round.png]

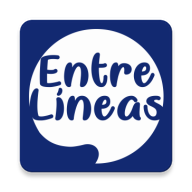

Supplement: Supplemental Information 1 [file peerj-cs-09-1223-s001.zip › BLApp-master/android/app/src/main/res/mipmap-xxxhdpi/ic_launcher.png]

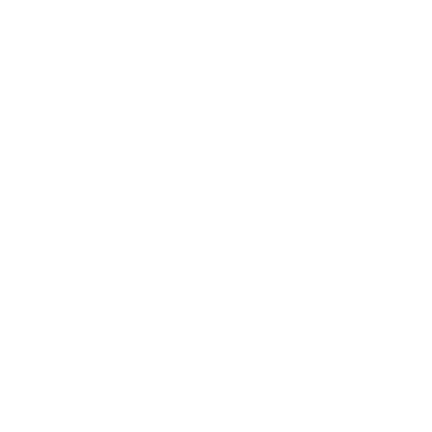

Supplement: Supplemental Information 1 [file peerj-cs-09-1223-s001.zip › BLApp-master/android/app/src/main/res/mipmap-xxxhdpi/ic_launcher_foreground.png]

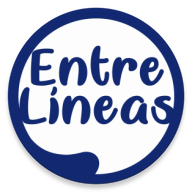

Supplement: Supplemental Information 1 [file peerj-cs-09-1223-s001.zip › BLApp-master/android/app/src/main/res/mipmap-xxxhdpi/ic_launcher_round.png]

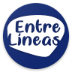

Supplement: Supplemental Information 1 [file peerj-cs-09-1223-s001.zip › BLApp-master/android/app/src/main/res/mipmap/ic_launcher.png]

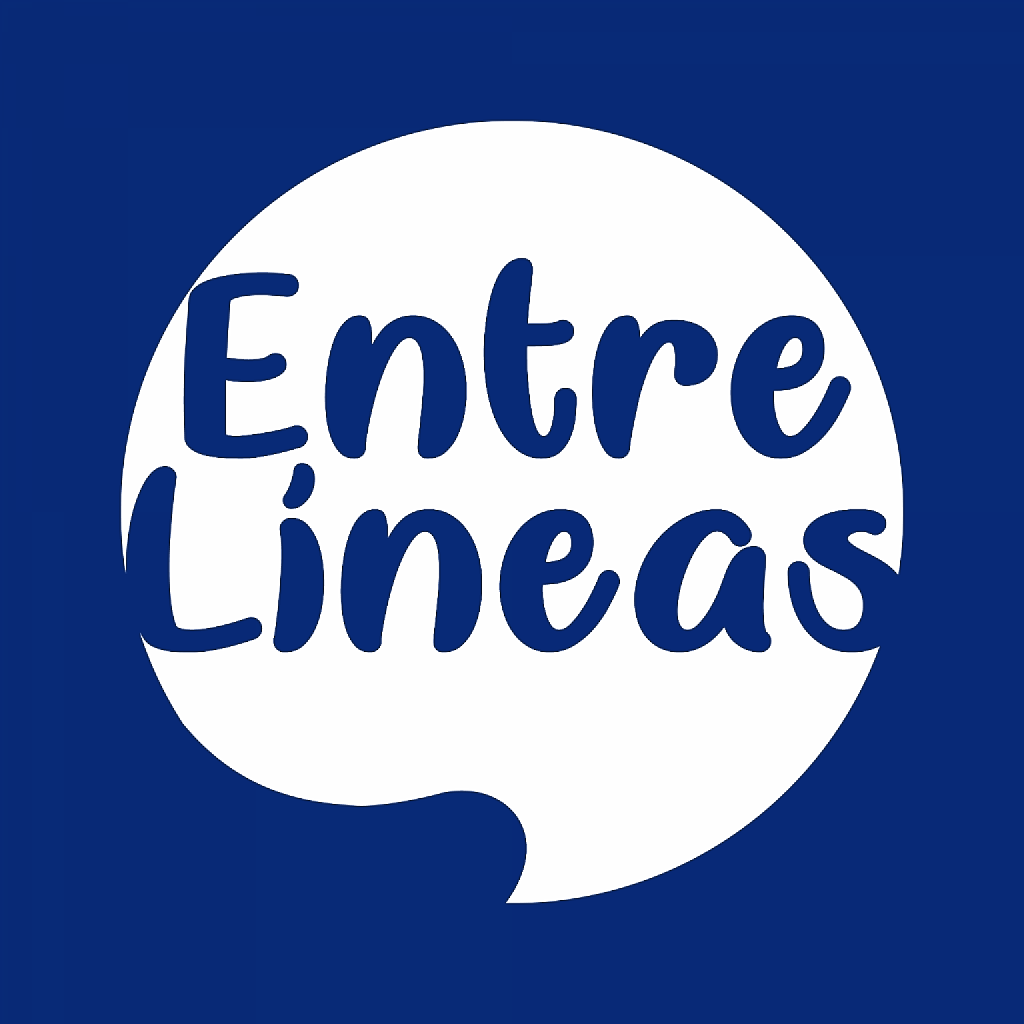

Supplement: Supplemental Information 1 [file peerj-cs-09-1223-s001.zip › BLApp-master/assets/AppIcons/Assets.xcassets/AppIcon.appiconset/1024.png]

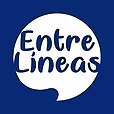

Supplement: Supplemental Information 1 [file peerj-cs-09-1223-s001.zip › BLApp-master/assets/AppIcons/Assets.xcassets/AppIcon.appiconset/114.png]

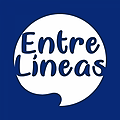

Supplement: Supplemental Information 1 [file peerj-cs-09-1223-s001.zip › BLApp-master/assets/AppIcons/Assets.xcassets/AppIcon.appiconset/120.png]

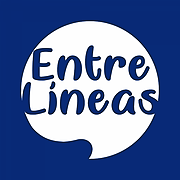

Supplement: Supplemental Information 1 [file peerj-cs-09-1223-s001.zip › BLApp-master/assets/AppIcons/Assets.xcassets/AppIcon.appiconset/180.png]

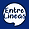

Supplement: Supplemental Information 1 [file peerj-cs-09-1223-s001.zip › BLApp-master/assets/AppIcons/Assets.xcassets/AppIcon.appiconset/29.png]

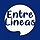

Supplement: Supplemental Information 1 [file peerj-cs-09-1223-s001.zip › BLApp-master/assets/AppIcons/Assets.xcassets/AppIcon.appiconset/40.png]

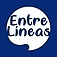

Supplement: Supplemental Information 1 [file peerj-cs-09-1223-s001.zip › BLApp-master/assets/AppIcons/Assets.xcassets/AppIcon.appiconset/57.png]

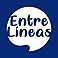

Supplement: Supplemental Information 1 [file peerj-cs-09-1223-s001.zip › BLApp-master/assets/AppIcons/Assets.xcassets/AppIcon.appiconset/58.png]

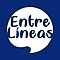

Supplement: Supplemental Information 1 [file peerj-cs-09-1223-s001.zip › BLApp-master/assets/AppIcons/Assets.xcassets/AppIcon.appiconset/60.png]

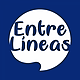

Supplement: Supplemental Information 1 [file peerj-cs-09-1223-s001.zip › BLApp-master/assets/AppIcons/Assets.xcassets/AppIcon.appiconset/80.png]

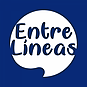

Supplement: Supplemental Information 1 [file peerj-cs-09-1223-s001.zip › BLApp-master/assets/AppIcons/Assets.xcassets/AppIcon.appiconset/87.png]

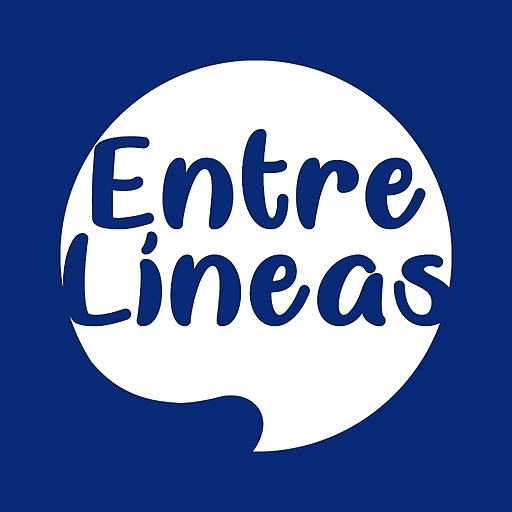

Supplement: Supplemental Information 1 [file peerj-cs-09-1223-s001.zip › BLApp-master/assets/AppIcons/playstore.png]

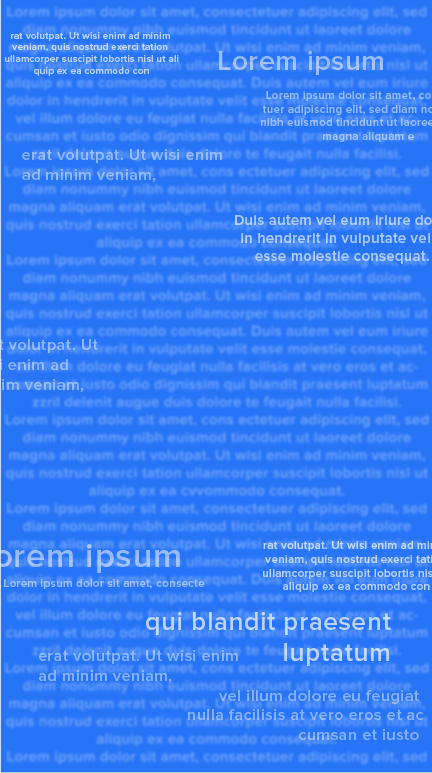

Supplement: Supplemental Information 1 [file peerj-cs-09-1223-s001.zip › BLApp-master/assets/Grafica Background 2.png]

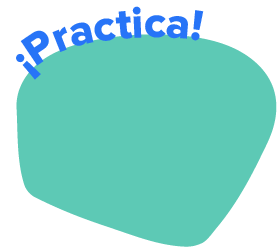

Supplement: Supplemental Information 1 [file peerj-cs-09-1223-s001.zip › BLApp-master/assets/Grafica Cuadro de practica.png]

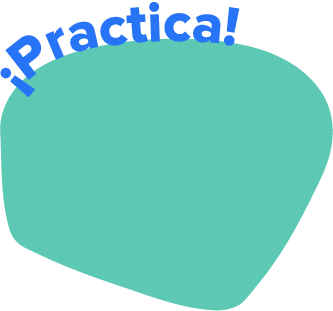

Supplement: Supplemental Information 1 [file peerj-cs-09-1223-s001.zip › BLApp-master/assets/Grafica Cuadro de practico.png]

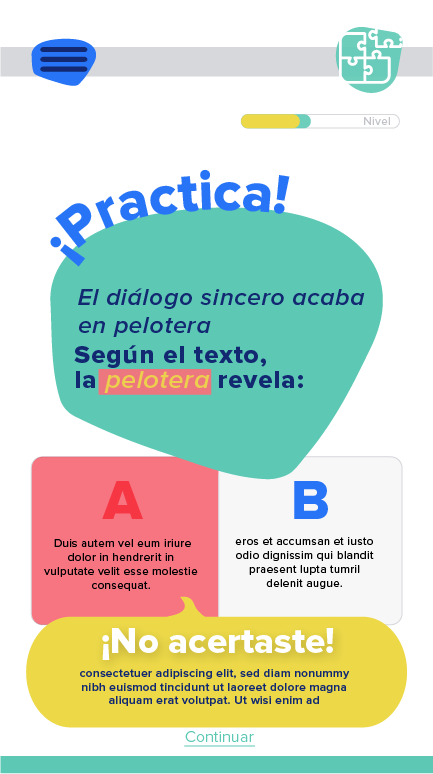

Supplement: Supplemental Information 1 [file peerj-cs-09-1223-s001.zip › BLApp-master/assets/Grafica EP - respuesta Incorrecta.png]

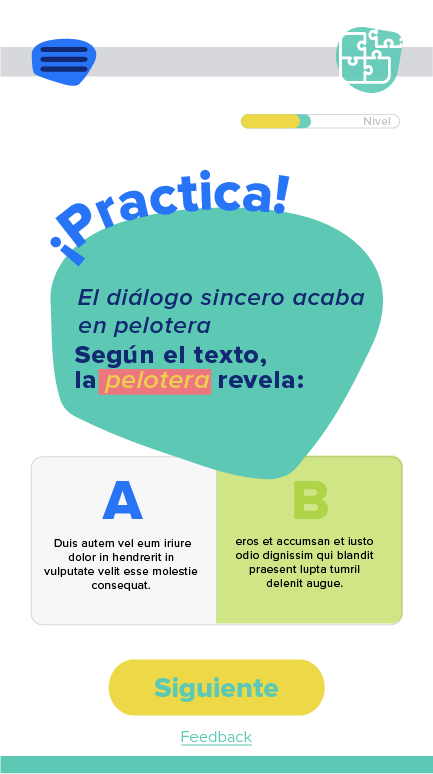

Supplement: Supplemental Information 1 [file peerj-cs-09-1223-s001.zip › BLApp-master/assets/Grafica EP - respuesta correcta.png]

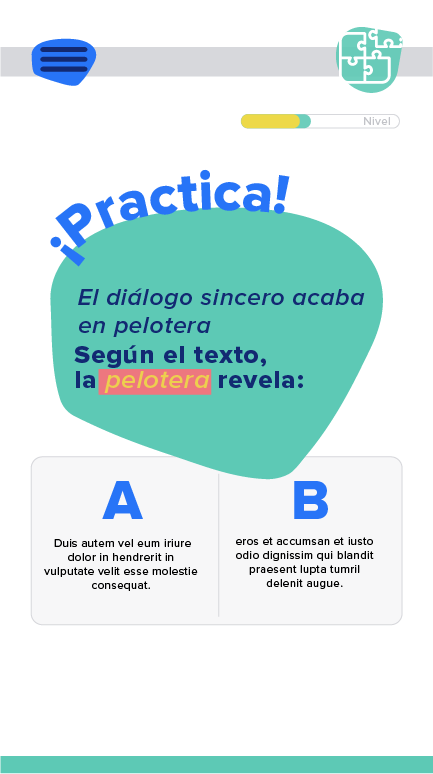

Supplement: Supplemental Information 1 [file peerj-cs-09-1223-s001.zip › BLApp-master/assets/Grafica Entorno de práctica.png]

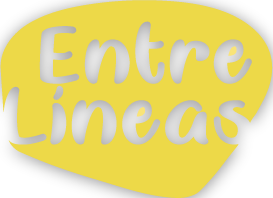

Supplement: Supplemental Information 1 [file peerj-cs-09-1223-s001.zip › BLApp-master/assets/Grafica Logo amarillo.png]

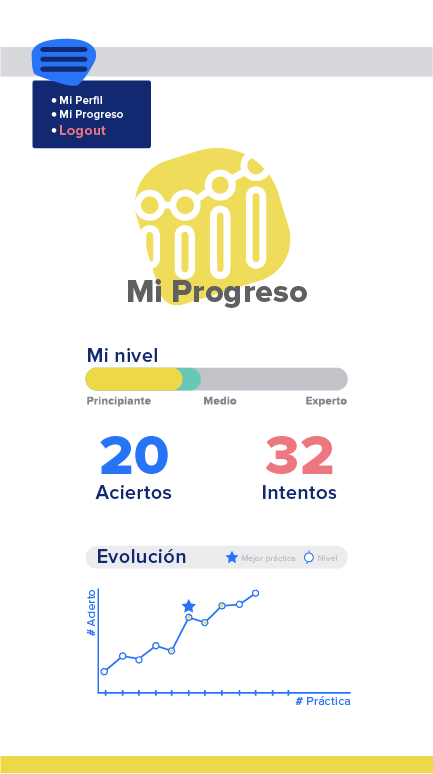

Supplement: Supplemental Information 1 [file peerj-cs-09-1223-s001.zip › BLApp-master/assets/Grafica Progress Screen.png]

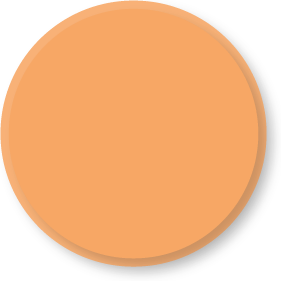

Supplement: Supplemental Information 1 [file peerj-cs-09-1223-s001.zip › BLApp-master/assets/Grafica Recuento de nivel fondo.png]

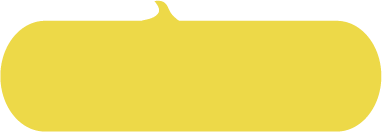

Supplement: Supplemental Information 1 [file peerj-cs-09-1223-s001.zip › BLApp-master/assets/feedback.png]

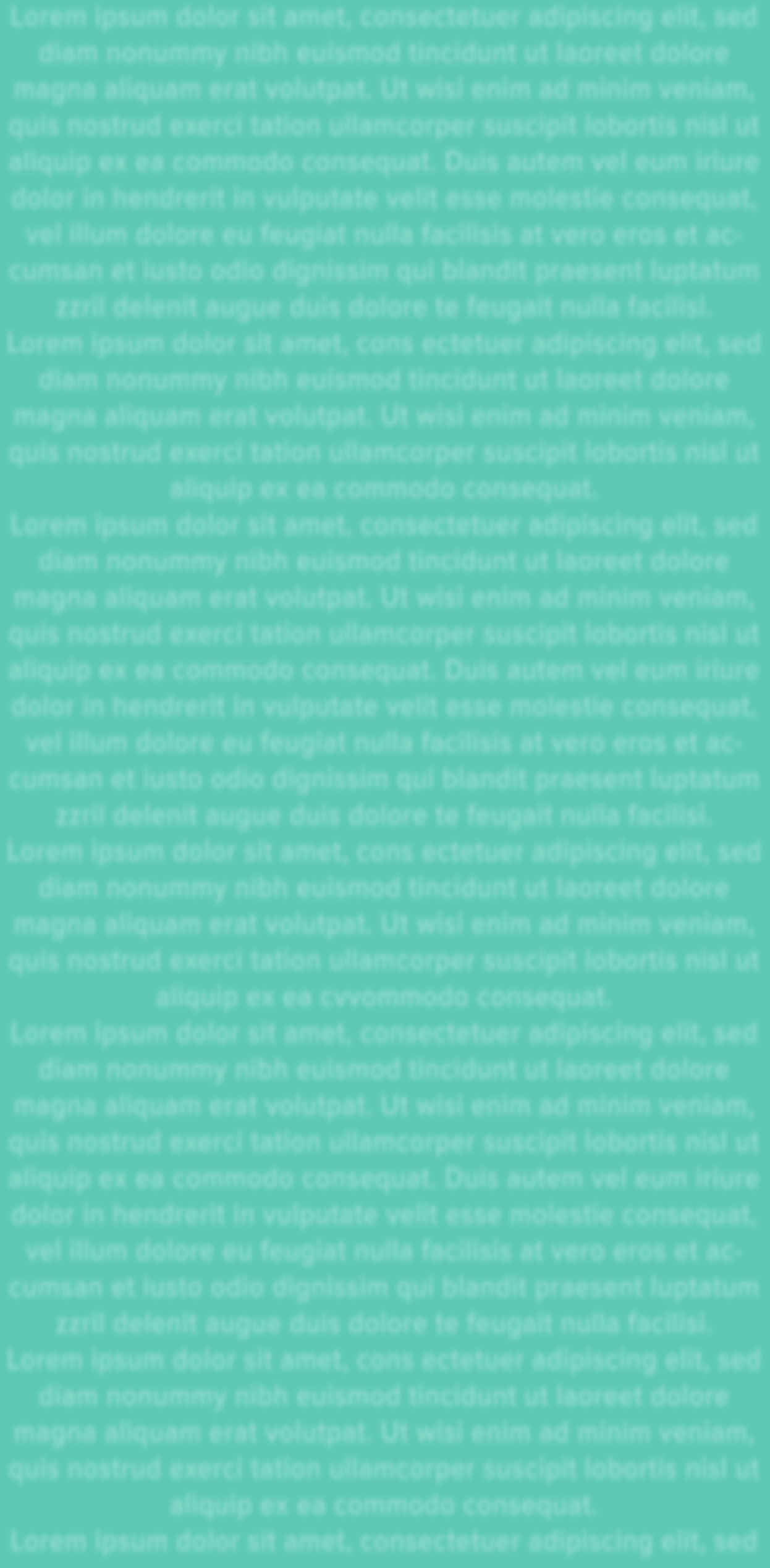

Supplement: Supplemental Information 1 [file peerj-cs-09-1223-s001.zip › BLApp-master/assets/fondo_azul.png]

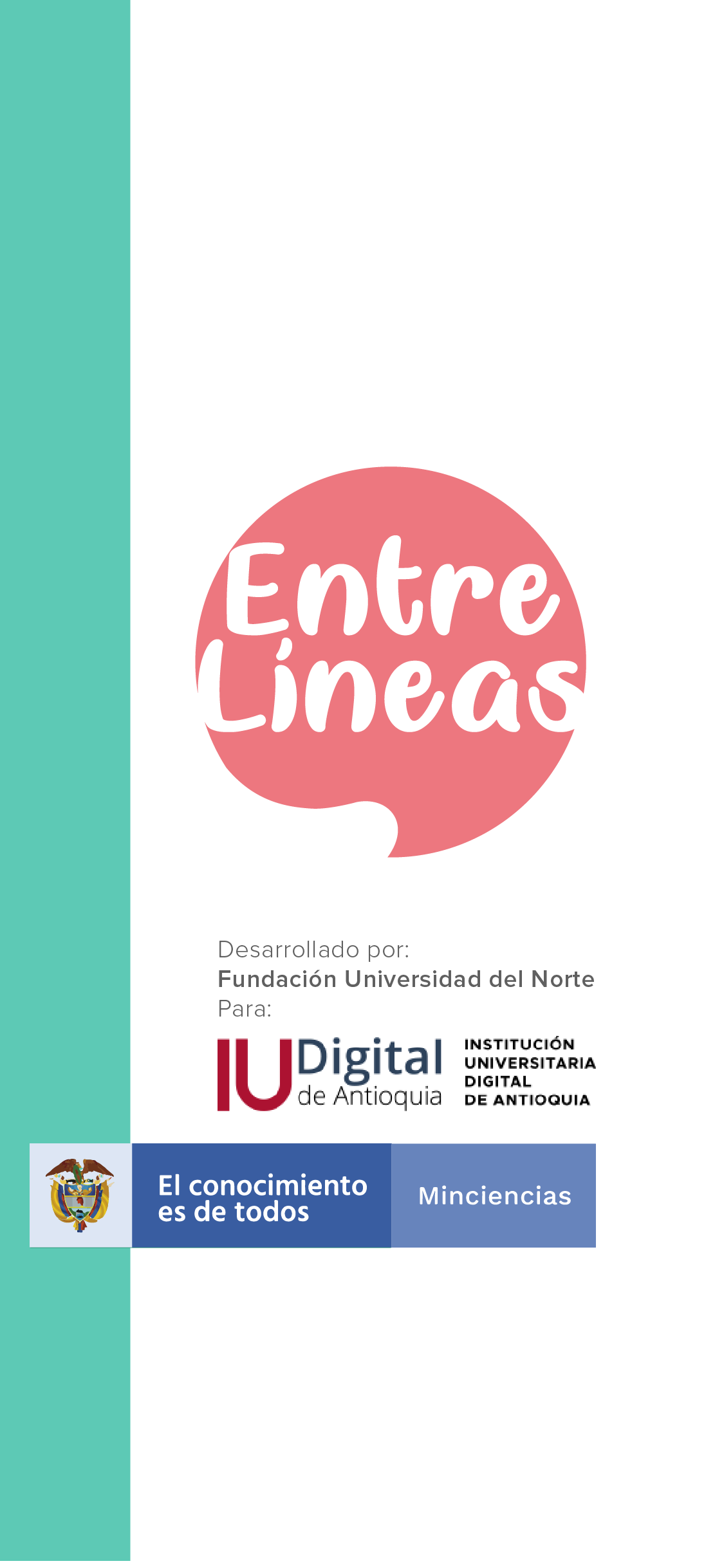

Supplement: Supplemental Information 1 [file peerj-cs-09-1223-s001.zip › BLApp-master/assets/fondo_colciencias.png]

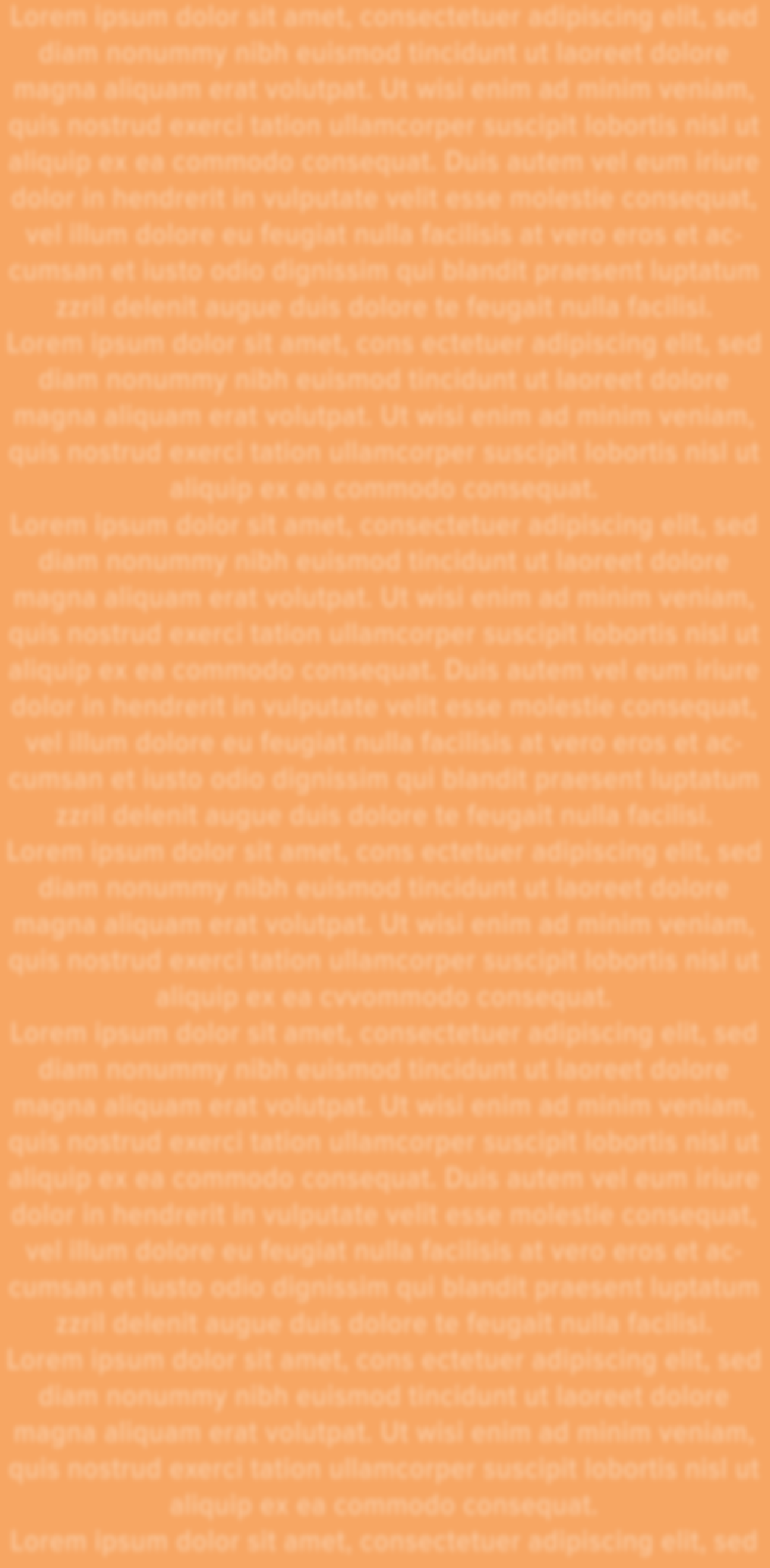

Supplement: Supplemental Information 1 [file peerj-cs-09-1223-s001.zip › BLApp-master/assets/fondo_naranja.png]

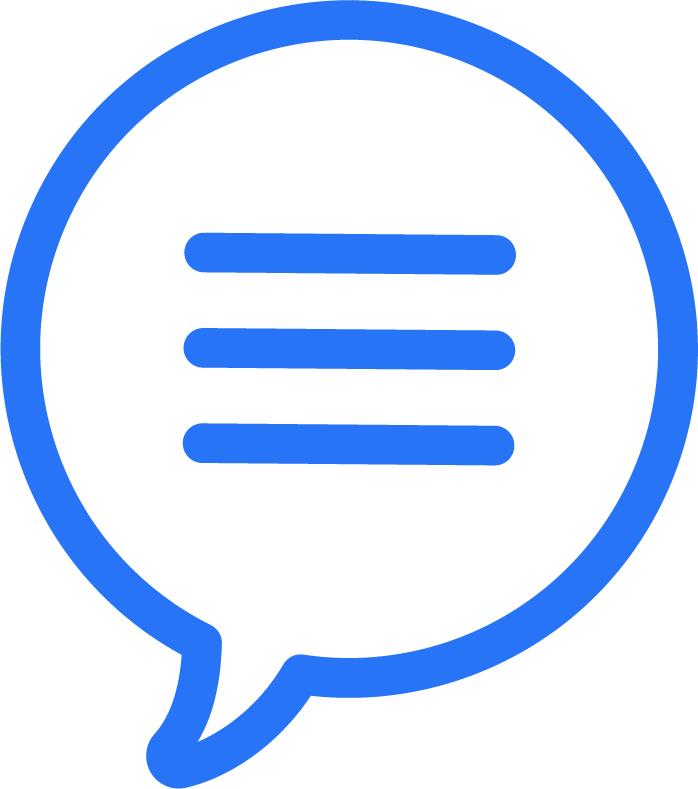

Supplement: Supplemental Information 1 [file peerj-cs-09-1223-s001.zip › BLApp-master/assets/foro_icon.png]

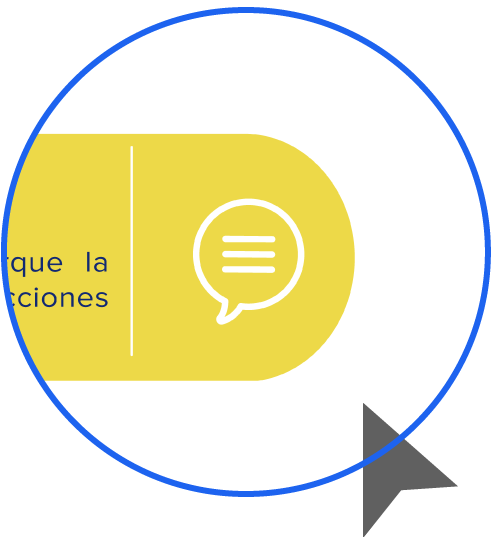

Supplement: Supplemental Information 1 [file peerj-cs-09-1223-s001.zip › BLApp-master/assets/forot.png]

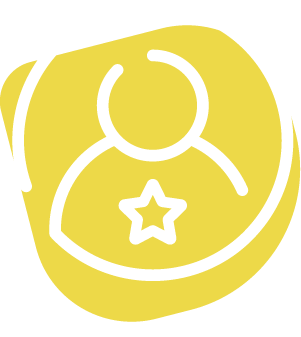

Supplement: Supplemental Information 1 [file peerj-cs-09-1223-s001.zip › BLApp-master/assets/icon_user_wt.png]

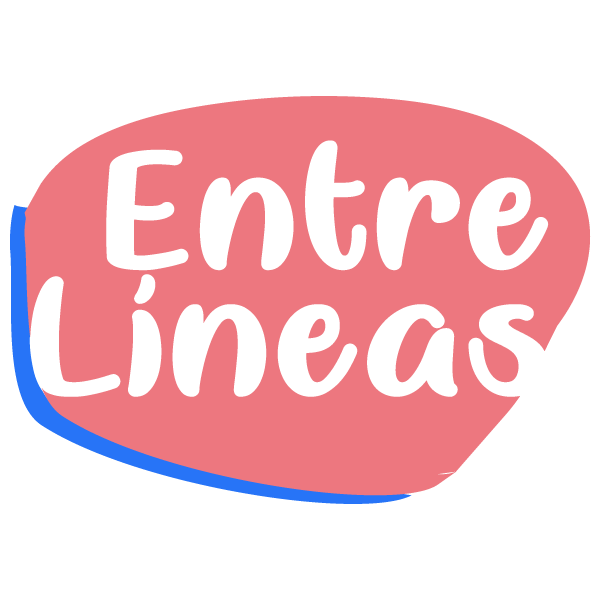

Supplement: Supplemental Information 1 [file peerj-cs-09-1223-s001.zip › BLApp-master/assets/logo.png]

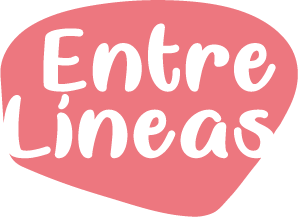

Supplement: Supplemental Information 1 [file peerj-cs-09-1223-s001.zip › BLApp-master/assets/logo_pink_white.png]

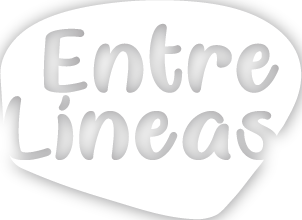

Supplement: Supplemental Information 1 [file peerj-cs-09-1223-s001.zip › BLApp-master/assets/logo_video.png]

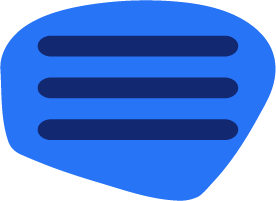

Supplement: Supplemental Information 1 [file peerj-cs-09-1223-s001.zip › BLApp-master/assets/menu.png]

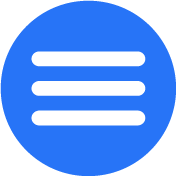

Supplement: Supplemental Information 1 [file peerj-cs-09-1223-s001.zip › BLApp-master/assets/menu_new_blue.png]

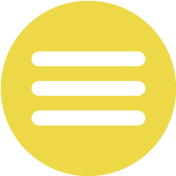

Supplement: Supplemental Information 1 [file peerj-cs-09-1223-s001.zip › BLApp-master/assets/menu_new_yellow.png]

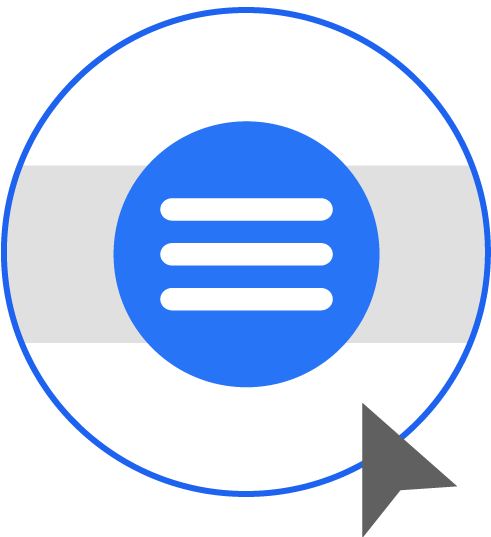

Supplement: Supplemental Information 1 [file peerj-cs-09-1223-s001.zip › BLApp-master/assets/menut.png]

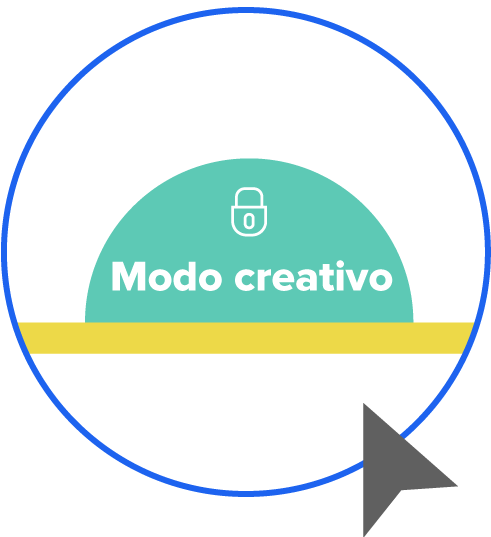

Supplement: Supplemental Information 1 [file peerj-cs-09-1223-s001.zip › BLApp-master/assets/modoct.png]

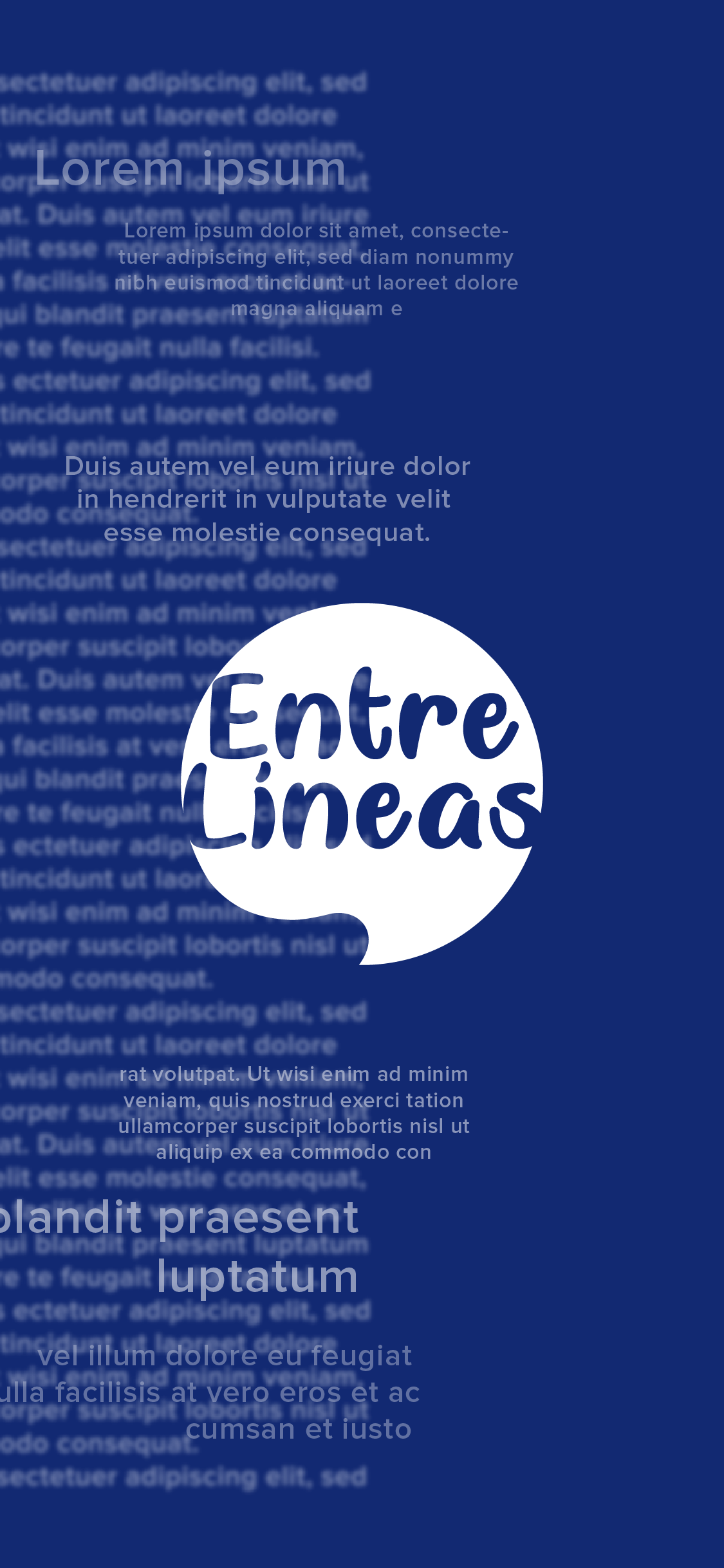

Supplement: Supplemental Information 1 [file peerj-cs-09-1223-s001.zip › BLApp-master/assets/nuevo_fondo.png]

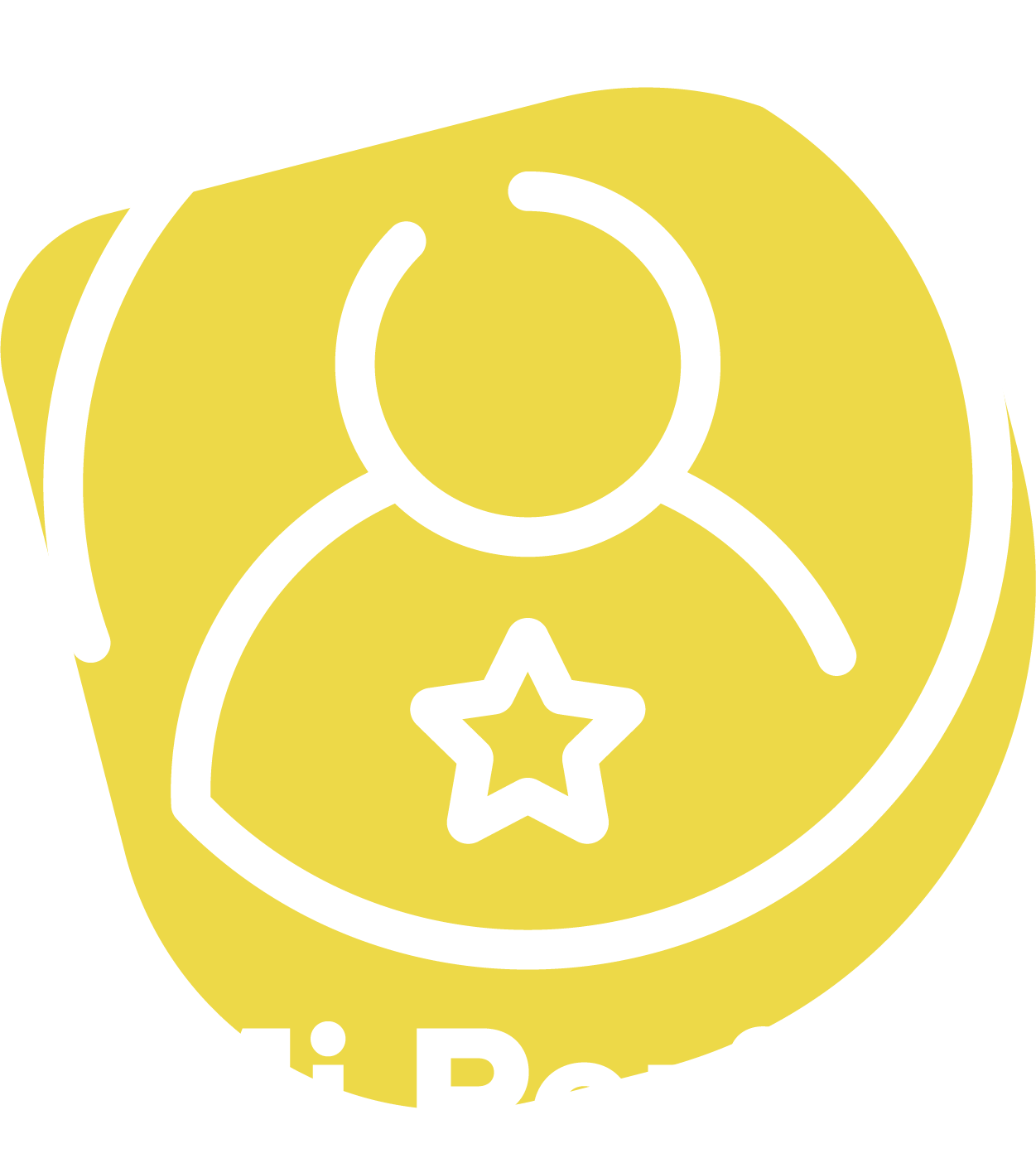

Supplement: Supplemental Information 1 [file peerj-cs-09-1223-s001.zip › BLApp-master/assets/perfil.png]

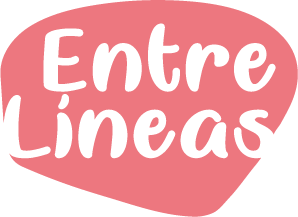

Supplement: Supplemental Information 1 [file peerj-cs-09-1223-s001.zip › BLApp-master/assets/pink_logo.png]

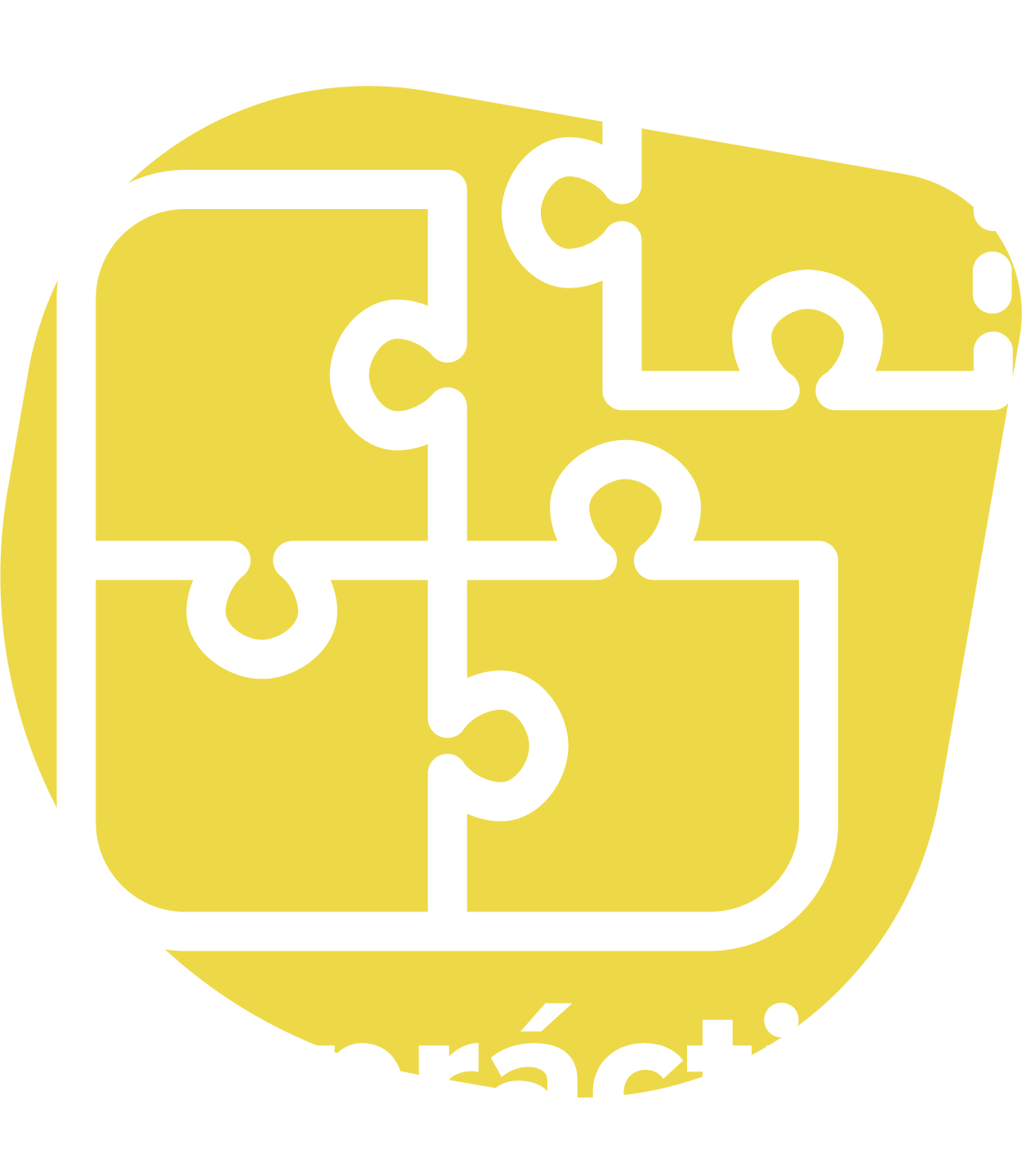

Supplement: Supplemental Information 1 [file peerj-cs-09-1223-s001.zip › BLApp-master/assets/practica.png]

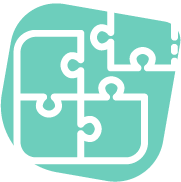

Supplement: Supplemental Information 1 [file peerj-cs-09-1223-s001.zip › BLApp-master/assets/practica_header.png]

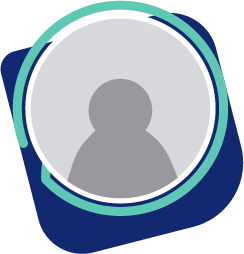

Supplement: Supplemental Information 1 [file peerj-cs-09-1223-s001.zip › BLApp-master/assets/profile.png]

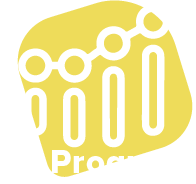

Supplement: Supplemental Information 1 [file peerj-cs-09-1223-s001.zip › BLApp-master/assets/progreso.png]

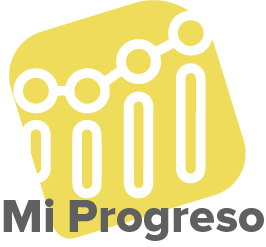

Supplement: Supplemental Information 1 [file peerj-cs-09-1223-s001.zip › BLApp-master/assets/progreso_black.png]

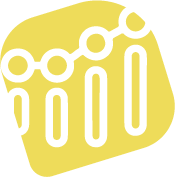

Supplement: Supplemental Information 1 [file peerj-cs-09-1223-s001.zip › BLApp-master/assets/progreso_header.png]

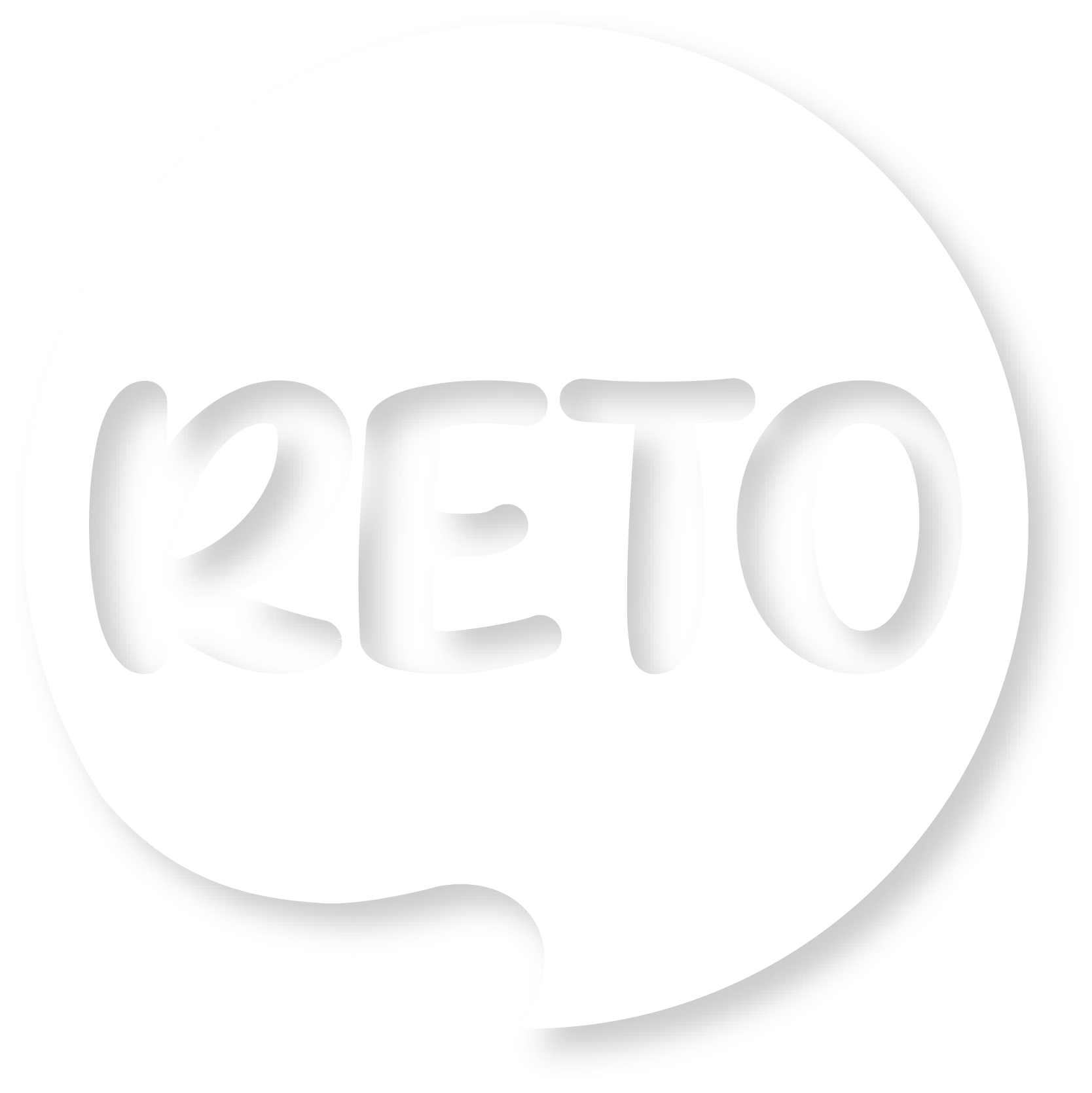

Supplement: Supplemental Information 1 [file peerj-cs-09-1223-s001.zip › BLApp-master/assets/reto.png]

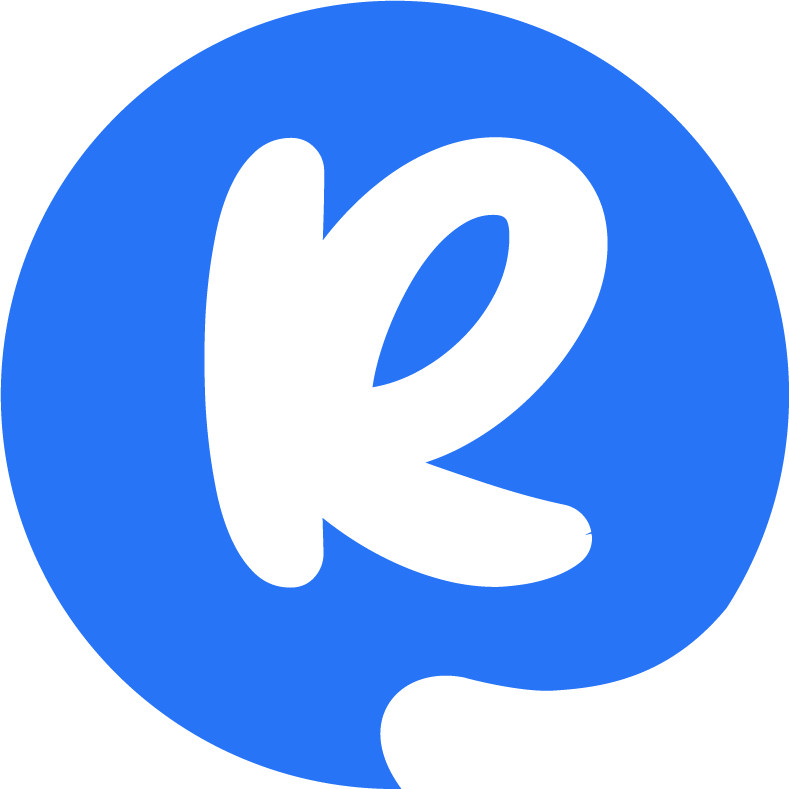

Supplement: Supplemental Information 1 [file peerj-cs-09-1223-s001.zip › BLApp-master/assets/reto_icon.png]

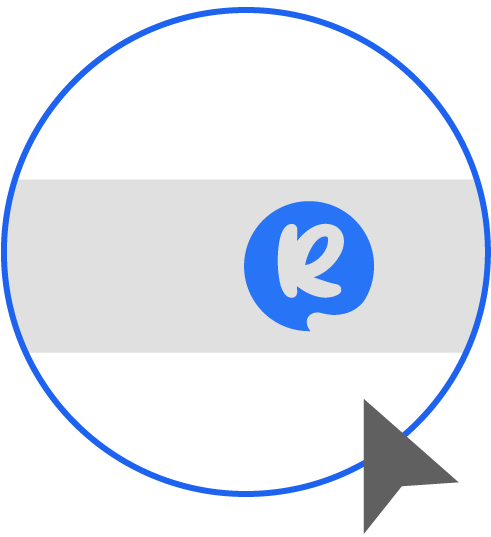

Supplement: Supplemental Information 1 [file peerj-cs-09-1223-s001.zip › BLApp-master/assets/retoicont.png]

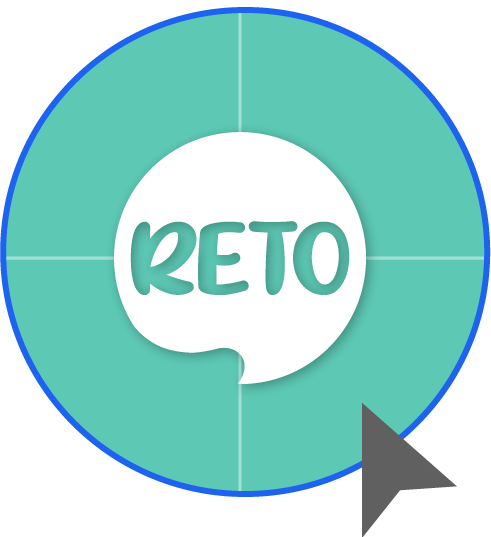

Supplement: Supplemental Information 1 [file peerj-cs-09-1223-s001.zip › BLApp-master/assets/retot.png]

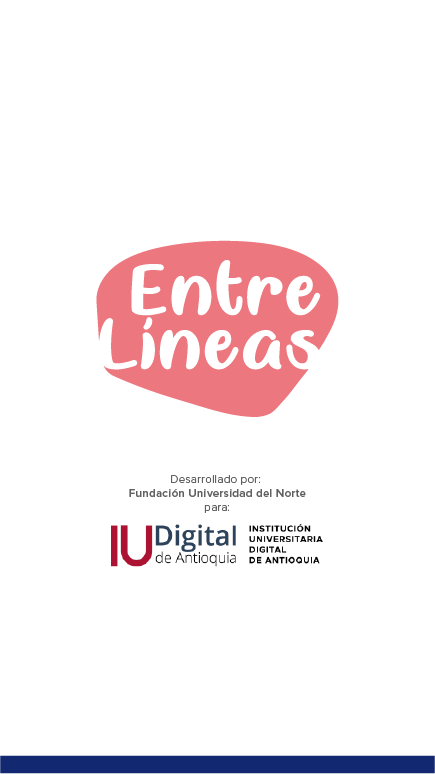

Supplement: Supplemental Information 1 [file peerj-cs-09-1223-s001.zip › BLApp-master/assets/splash.png]

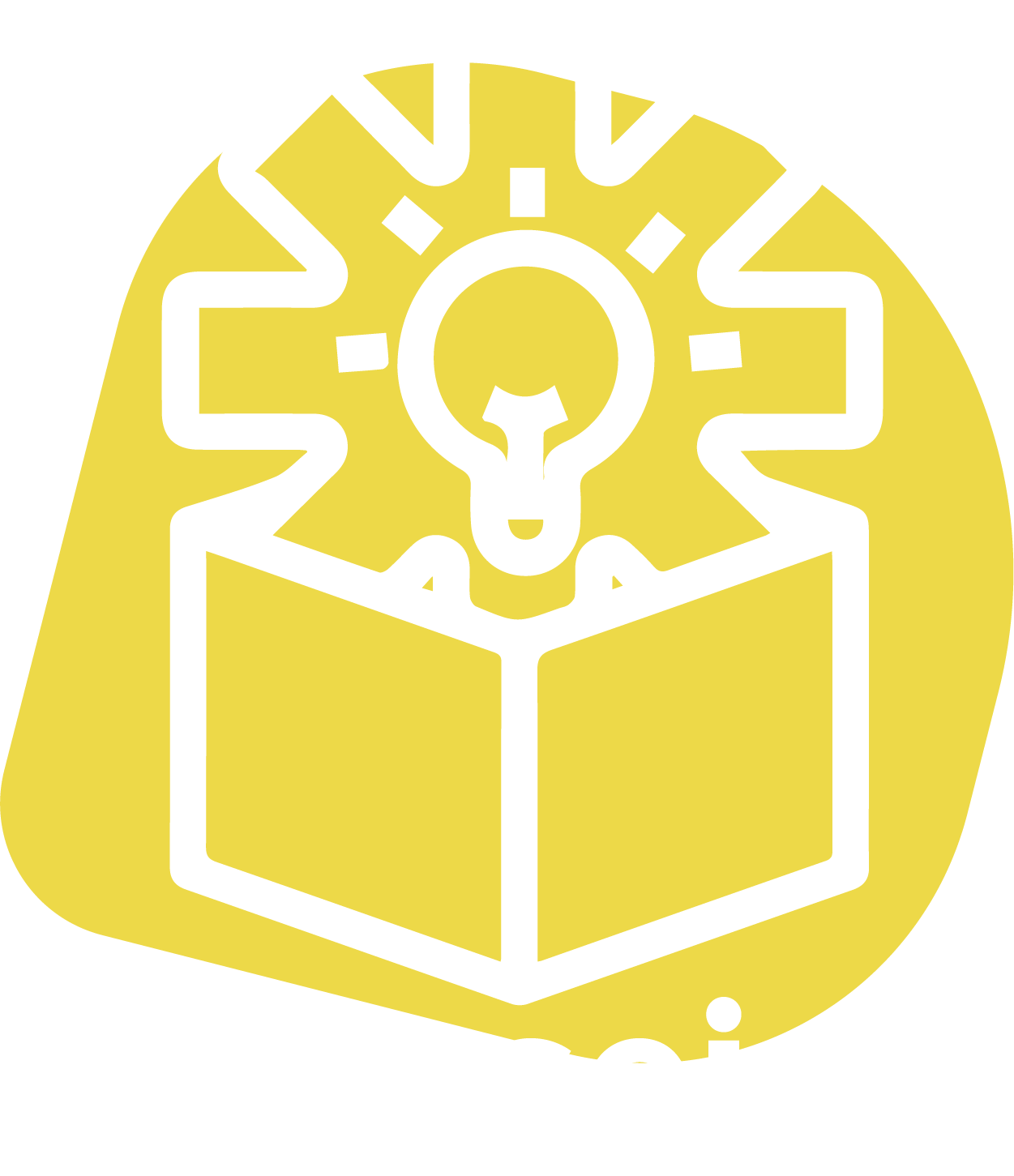

Supplement: Supplemental Information 1 [file peerj-cs-09-1223-s001.zip › BLApp-master/assets/tips.png]

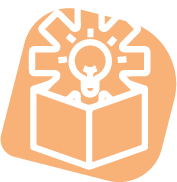

Supplement: Supplemental Information 1 [file peerj-cs-09-1223-s001.zip › BLApp-master/assets/tips_header.png]

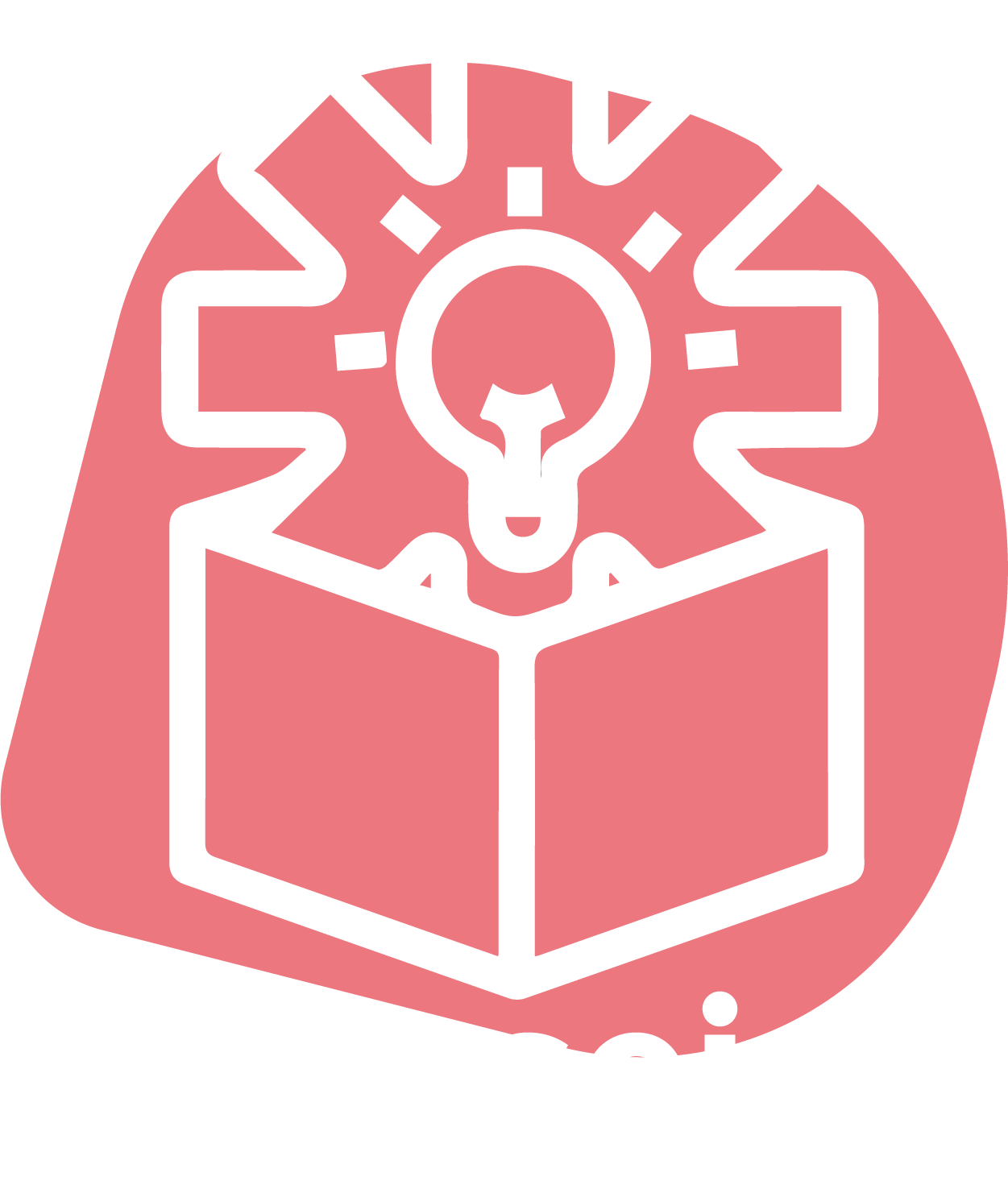

Supplement: Supplemental Information 1 [file peerj-cs-09-1223-s001.zip › BLApp-master/assets/tips_pink.png]

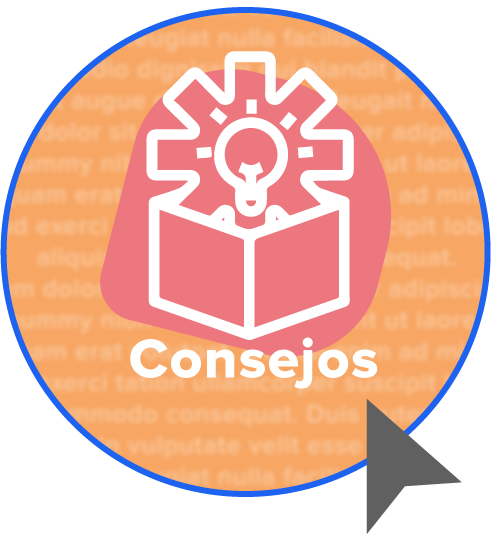

Supplement: Supplemental Information 1 [file peerj-cs-09-1223-s001.zip › BLApp-master/assets/tipst.png]

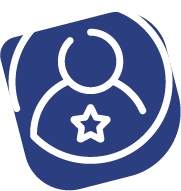

Supplement: Supplemental Information 1 [file peerj-cs-09-1223-s001.zip › BLApp-master/assets/user_blue.png]

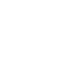

Supplement: Supplemental Information 1 [file peerj-cs-09-1223-s001.zip › BLApp-master/assets/watch.png]

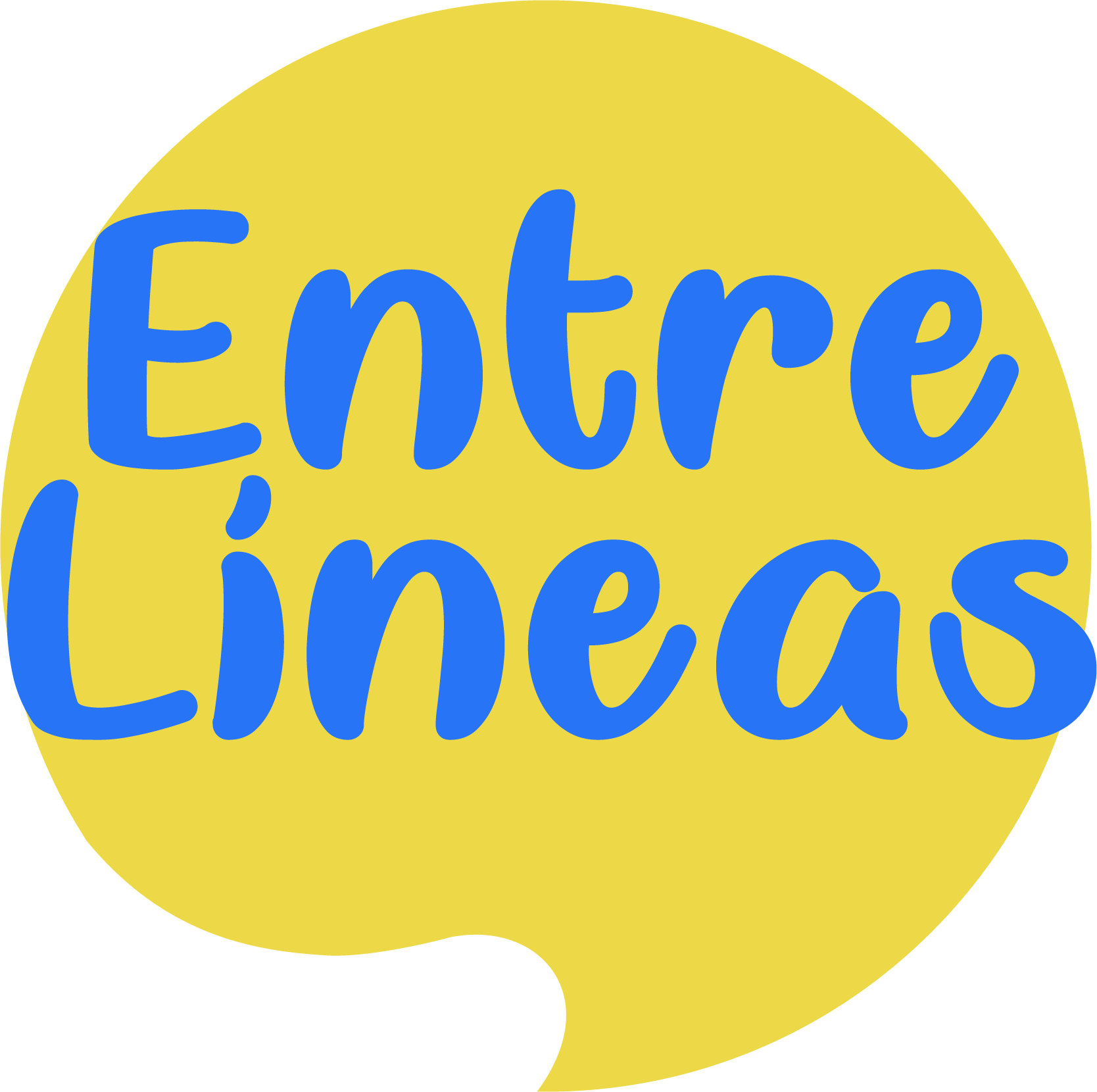

Supplement: Supplemental Information 1 [file peerj-cs-09-1223-s001.zip › BLApp-master/assets/yn_logo.png]

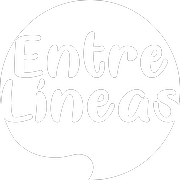

Supplement: Supplemental Information 1 [file peerj-cs-09-1223-s001.zip › BLApp-master/ios/BLApp/Images.xcassets/SplashIcon.imageset/Icon-60x60@3x.png]

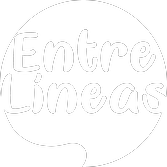

Supplement: Supplemental Information 1 [file peerj-cs-09-1223-s001.zip › BLApp-master/ios/BLApp/Images.xcassets/SplashIcon.imageset/Icon-83.5@2x.png]

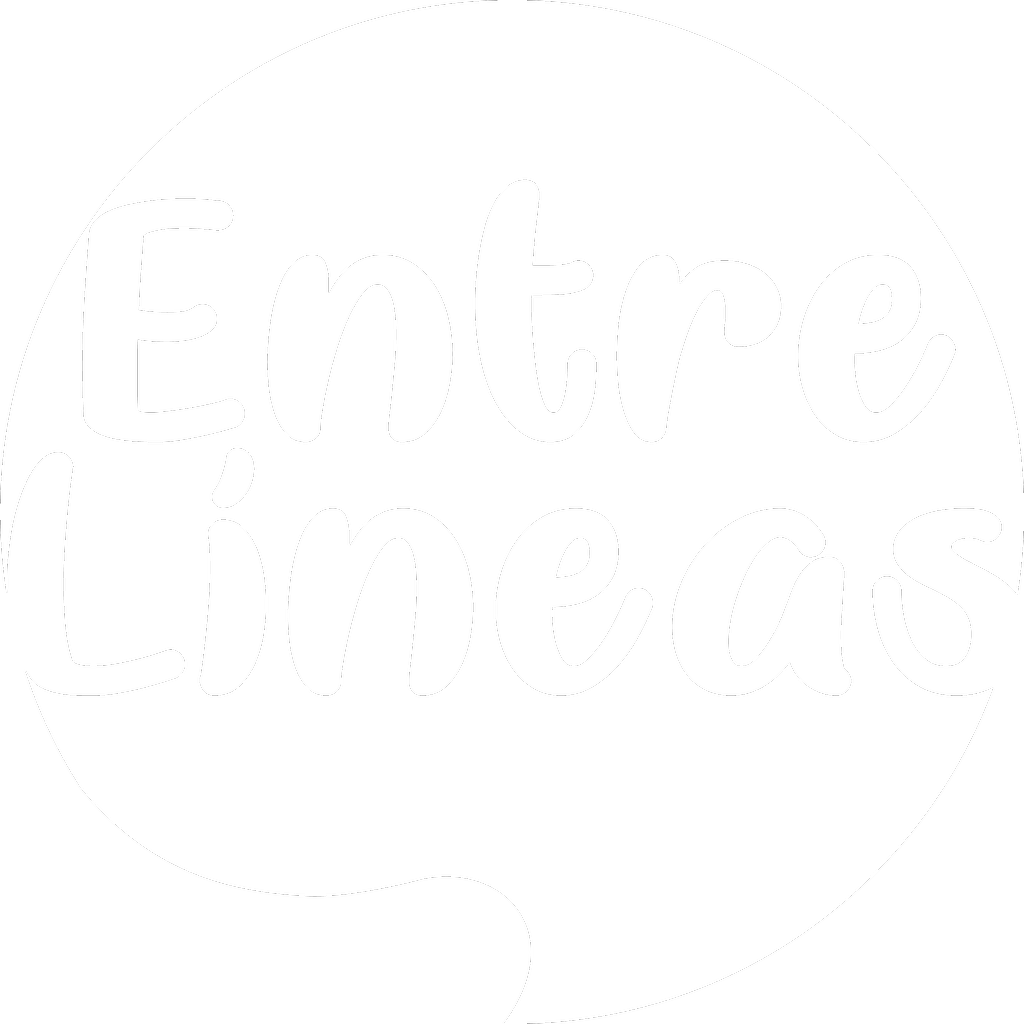

Supplement: Supplemental Information 1 [file peerj-cs-09-1223-s001.zip › BLApp-master/ios/BLApp/Images.xcassets/SplashIcon.imageset/Icon-marketing-1024x1024.png]
